# An experimental method to study emissions from heated tobacco between 100-200°C

**DOI:** 10.1186/s13065-015-0096-1

**Published:** 2015-04-16

**Authors:** Mark Forster, Chuan Liu, Martin G Duke, Kevin G McAdam, Christopher J Proctor

**Affiliations:** GR&D Centre, British American Tobacco, Regents Park Road, Southampton, SO15 8TL UK

**Keywords:** Tobacco heating, Aerosol, Emission, Toxicant

## Abstract

**Background:**

Cigarette smoke emissions are mainly produced by distillation, pyrolysis and combustion reactions when the tobacco is burnt. Some studies have shown that heating tobacco to temperatures below pyrolysis and combustion temperatures has the potential to reduce or eliminate some toxicants found in cigarette smoke. In this study, we designed a bench-top tube furnace that heats tobacco between 100-200°C and systematically studied the effects of heating temperatures on selected gas phase and aerosol phase compounds using an ISO machine-smoking protocol.

**Results:**

Among a list of target chemical compounds, seven toxicants (nicotine, carbon monoxide, acetaldehyde, crotonaldehyde, formaldehyde, NNN and NNK) were quantifiable but not at all temperatures examined. The levels of the compounds generally displayed an increasing trend with increasing temperatures. The observed carbon monoxide and aldehydes represented the initial thermal breakdown products from the tobacco constituents. Water was the largest measured component in the total aerosol phase collected and appeared to be mainly released by evaporation; nicotine release characteristics were consistent with bond breaking and evaporation. Quantifiable levels of NNK and NNN were thought to be the result of evaporative transfer from the tobacco blend.

**Conclusions:**

These results demonstrate the practical utility of this tool to study low-temperature toxicant formation and emission from heated tobacco. Between 100 to 200°C, nicotine and some cigarette smoke compounds were released as a result of evaporative transfer or initial thermal decomposition from the tobacco blend.

**Electronic supplementary material:**

The online version of this article (doi:10.1186/s13065-015-0096-1) contains supplementary material, which is available to authorized users.

## Background

Cigarette smoke is a highly complex aerosol system [1], involving over 6,000 identified chemicals in a dynamic and reactive mixture [2,3]. These chemicals are generated by incomplete combustion of tobacco that burns in either smouldering or puffing modes. Research efforts aimed at modifying combustion conditions with the aim of reducing smoke toxicity have so far proven difficult [4]. An alternative way to reduce the generation of toxicants from tobacco product is to heat rather than burn tobacco (sometimes referred to as heat-not-burn technologies). This has been tried in two ways. The first approach involves a lit carbon tip that heats incoming air, which in turn heats tobacco-based substrates in a form of cigarette-like product, forming an aerosol containing mainly water, glycerol, nicotine and volatile tobacco components [5,6]. Another approach uses a battery-powered smoking device containing a specially designed tobacco rod [7-11]. In both cases, the tobacco heating temperatures are typically below 300°C, enough to release nicotine but not high as to cause significant pyrolysis. Studies based on these two types of technology have generally shown that the aerosol composition is somewhat simpler than that found in cigarette smoke [6,11,12].

Studies to understand low-temperature tobacco thermochemistry have shown that the nicotine organic salts, the natural and stable form of nicotine in the tobacco leaf, begin to release nicotine above ca. 150°C [13-15]. Historically, pyrolysis studies on tobacco and tobacco ingredients have been performed around temperature ranges in the pyrolysis and combustion zones of the burning tip of a cigarette — typically above 500°C and up to 1000°C [16,17]. This is because most of the particulate phase components (collectively known as tar) in cigarette smoke are produced by thermal reactions within the tobacco rod at these temperatures. Many pyrolysis studies have examined both qualitative and quantitative relationships between different tobacco leaf and leaf ingredients (as possible precursors to smoke components) and smoke constituents of interests [18-25]. Data resulting from these studies have been useful in assessing whether or to what extent a specific ingredient or precursor will undergo thermal decomposition, their tendency to be transferred to the smoke intact, or possibly form a smoke constituent. In contrast, studies of the thermochemistry of tobacco heated to lower temperatures (under 300°C) are not common. The ones published are usually based on a specific product [11,12], hence their use in understanding aspects of low-temperature tobacco thermochemistry is limited. In this study, we examined levels of selected emissions in the aerosol produced by heating tobacco between 100 and 200°C to better understand aerosol properties from low-temperature tobacco heating. For this purpose, we developed a bench-top furnace for aerosol generation and collection from controlled tobacco heating. The focus of the investigation was on the low-temperature releasing mechanisms for some known compounds typically associated with tobacco.

## Results and discussion

### Thermogravimetric analysis (TGA)

**Figure 1:**
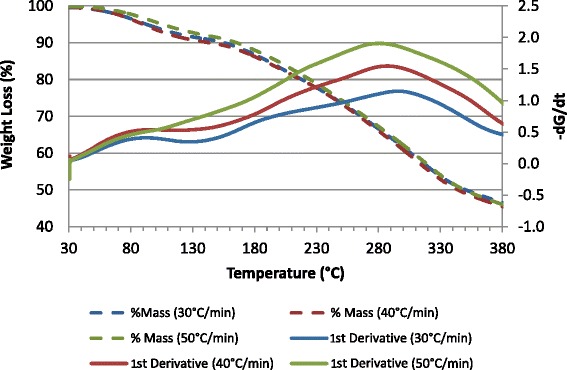
TGA of tobacco heated in air at three heating rates.

The TGA results up to 300°C are plotted in Figure 1. The rate of weight loss (−dG/dT) began with a significant weight loss up to 100°C due to the release of free or physically bound water, with a second major weight loss occurring over a relatively broad temperature range above ca. 200°C. The water loss around 100°C accounted for approximately 7% of the weight loss. At the end of 200°C, the samples had lost about 17% of their initial weight which was more than the 11% moisture content, suggesting thermal evaporation and possibly onset of some initial thermal decomposition of some tobacco constituents [16,18,19]. The heating rates used in Figure 1 are significantly slower than those typically found in a burning cigarette during a puff where the tobacco heating rate can exceed a few hundred degrees per second [1]. Within the range of heating rates studied, there was no discernable weight loss trend in Figure 1, however, the 1st derivative of the weight loss showed a temporary plateau around 100°C and a gentle reflection point between 280 to 320°C.

**Figure 2:**
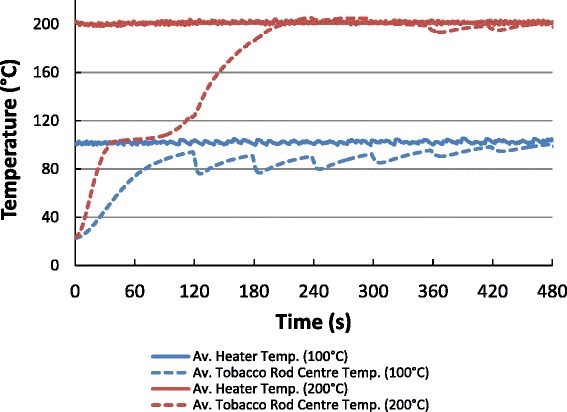
Temperature profiles of the heating chamber of the furnace (solid lines) and the centre of the tobacco rod (dashed lines) at 100 and 200°C.

Figure 2 shows the temperature responses from the heater and also the centre of the tobacco rod at two set heater temperatures, 100 and 200°C. Apart from minor fluctuations, the heater temperatures tracked the set temperature within ± 5°C. When the furnace was set at 100°C, the centre of the tobacco rod reached this temperature (within ~5%) after about 120 s. As the heat was supplied by conduction and radiation from the peripheral surface, this measurement point was the last part of the tobacco rod to achieve the target temperature. A puff at 120 s created a sudden and significant temperature drop, most likely due to the cooling effect by incoming air and nearly 60 s was required for the temperature to recover to 100°C. This temperature drop by puffing became progressively less significant in the later puffs, probably as a result of larger amount of energy stored within the tobacco rod combined with less amount of water available to release.

When the furnace was set at 200°C, the initial temperature gradient achieved by the rod centre was greater than that set at 100°C, representing a faster heat up of the rod. However, this temperature rise stopped abruptly around 100°C and remained there for nearly 60 s. This temporary temperature plateau not only occurred when the furnace was set at 200°C but also observed at the other set temperatures above 100°C (not shown), thus it can be attributed to the energy required to release the water from the tobacco rod, about 7% as measured by the TGA results in Figure 1. At the 200°C heater temperature, once the temperature rise proceeded beyond this plateau, the magnitude of the temperature drop caused by puffing was very small. The main thermophysical processes responsible for aerosol release from a peripherally heated tobacco are significantly different as those seen in a lit cigarette as described by Baker [1,4]. In Figure 2, the heat is supplied from the outer surface of the tobacco along the length of the rod and the aerosol produced during a puff is the result of the heating of the tobacco rod during the period prior to the puff. Puffing simply transports pre-formed aerosol outside of the tobacco rod.

### Aerosol composition from heated tobacco

Table 1 lists the emissions measured from the heated tobacco samples together with standard deviation values. Total particulate matter (TPM), water, nicotine and nicotine-free-dry-particulate-matter (NFDPM) have also been included. The term NFDPM is a convention used to describe the condense phase material of cigarette smoke. No systematic studies have been published at the present time to account for the full emission composition produced by heating tobacco at lower temperatures. This work chose to focus on a number of known cigarette smoke toxicants, as they cover permanent gases, typical vapour phase and aerosol phase compounds, as well as evaporatively and pyrolytically generated substances. The relatively large standard deviation values reflect the fact these analytical procedures need improvement and optimisation for the different emission matrix from the heated tobacco.

**Table 1:** **Amounts of toxicants (mean level ± SD, n = 3) in the aerosol from heated tobacco at six different temperatures**

	**unit**	**Tobacco Heating Temperature (°C)**					
		**100**	**120**	**140**	**160**	**180**	**200**
TPM	mg/sample	11.05 ± 2.14	13.55 ± 4.69	23.31 ± 6.90	19.43 ± 6.79	30.47 ± 5.10	32.07 ± 6.20
Water	mg/sample	8.41 ± 1.88	10.13 ± 3.99	14.47 ± 0.64	13.11 ± 3.97	16.73 ± 1.02	18.81 ± 1.71
Nicotine	mg/sample	<0.10	<0.10	0.21 ± 0.11	0.45 ± 0.28	1.28 ± 0.39	1.55 ± 0.72
NFDPM	mg/sample	2.62 ± 0.29	3.38 ± 0.70	8.64 ± 6.21	5.88 ± 2.83	12.46 ± 3.78	11.71 ± 6.47
Ammonia	μg/sample	<0.80	<0.80	<0.80	<0.80	<0.80	<0.80
Carbon monoxide	mg/sample	0.01 ± 0.02	0.00 ± 0.00	0.01 ± 0.02	0.01 ± 0.02	0.07 ± 0.04	0.13 ± 0.02
Acetaldehyde	μg/sample	<1.20	6.5 ± 1.3	29.8 ± 4.7	61.9 ± 6.3	78.4 ± 1.9	84.6 ± 15.1
Acrolein	μg/sample	<1.00	<1.00	<1.00	<1.00	<1.00	<1.00
Crotonaldehyde	μg/sample	<1.10	<1.10	<1.10	<1.10	2.0 ± 0.1	4.1 ± 0.2
Formaldehyde	μg/sample	<0.90	<0.90	<0.90	<0.90	1.0 ± 0.1	2.9 ± 1.2
Hydrogen cyanide	μg/sample	<5.60	<5.60	<5.60	<5.60	<5.60	<5.60
Catechol	μg/sample	<4.00	<4.00	<4.00	<4.00	<4.00	<4.00
*m-*Cresol	μg/sample	<0.40	<0.40	<0.40	<0.40	<0.40	<0.40
*o-*Cresol	μg/sample	<0.40	<0.40	<0.40	<0.40	<0.40	<0.40
*p-*Cresol	μg/sample	<0.40	<0.40	<0.40	<0.40	<0.40	<0.40
Hydroquinone	μg/sample	<3.20	<3.20	<3.20	<3.20	<3.20	<3.20
Phenol	μg/sample	<1.60	<1.60	<1.60	<1.60	<1.60	<1.60
NNN	ng/sample	0.8 ± 0.2	1.4 ± 0.3	2.3 ± 0.7	1.8 ± 0.6	1.5 ± 0.8	1.8 ± 0.8
NNK	ng/sample	0.7 ± 0.1	0.9 ± 0.1	1.7 ± 0.5	1.3 ± 0.7	1.4 ± 1.0	0.7 ± 0.1
NAB	ng/sample	<0.18	<0.18	0.3 ± 0.1	0.2 ± 0.1	0.4 ± 0.0	0.4 ± 0.3
NAT	ng/sample	0.4 ± 0.1	0.8 ± 0.2	1.3 ± 0.6	1.1 ± 0.6	1.5 ± 1.4	4.2 ± 3.2
Acrylonitrile	μg/sample	<0.33	<0.33	<0.33	<0.33	<0.33	<0.33
Benzene	μg/sample	<1.42	<1.42	<1.42	<1.42	<1.42	<1.42
1,3-Butadiene	μg/sample	<2.02	<2.02	<2.02	<2.02	<2.02	<2.02
Isoprene	μg/sample	<8.25	<8.25	<8.25	<8.25	<8.25	<8.25
Toluene	μg/sample	<2.10	<2.10	<2.10	<2.10	<2.10	<2.10
Acetone	μg/sample	<1.50	<1.50	<1.50	3.6 ± 1.1	7.0 ± 0.5	8.4 ± 3.7
Butyraldehyde	μg/sample	<1.20	<1.20	5.4 ± 0.7	10.5 ± 0.9	10.9 ± 0.7	9.5 ± 2.2
Methyl ethyl ketone	μg/sample	<1.20	<1.20	<1.20	<1.20	1.3 ± 0.0	1.9 ± 0.5
Propionaldehyde	μg/sample	<0.95	<0.95	<0.95	1.7 ± 0.3	3.5 ± 0.2	5.6 ± 0.8
Resourcinol	μg/sample	<0.40	<0.40	<0.40	<0.40	<0.40	<0.40

For comparison purpose, the mainstream smoke yields of the cigarette is given in the Additional file 1.

For temperatures under 140°C, nicotine deliveries were below the reporting limit of the analytical method (0.1 mg/sample). Consistent with this, previous studies show that nicotine or nicotine salts begin measurable weight losses above 150°C [14]. Nicotine delivery increased rapidly between 160 and 180°C, again in agreement with the other studies. Across the temperature range, there was also an increase in TPM, water and NFDPM levels. A significant amount of the TPM was made of water, nevertheless a measurable mass of tobacco constituents were released at 100°C and above. Carbon monoxide yields were below the reporting limits below 180°C. Above this temperature (to 200°C), carbon monoxide levels increased with increasing temperatures. During the experiments, no blanks were run to account for the atmospheric carbon monoxide levels. Natural background carbon monoxide concentration has been reported to be of 0.04 ppm [26], thus the reporting limit of 0.01 mg/cig (approximately 100 ppm) from this work is too high to show any contribution from environmental carbon monoxide. Baker’s study on cellulose and tobacco [16] pointed out that carbon monoxide was formed mainly by a low-temperature decomposition of tobacco constituents around and above 180°C.

The levels of other analytes also increased gradually with rising temperature, including acetaldehyde (120 to 200°C), crotonaldehyde (180 to 200°C), formaldehyde (180 to 200°C), acetone (160 to 200°C), butyraldehyde (140 to 200°C), methyl ethyl ketone (180 to 200°C) and propionaldehyde (160 to 200°C). Trace amounts of these compounds have been reported in smokeless tobacco products [27], but the volatility of these compounds means that were they to contribute to the quantities measured in this work then they would be observed at all temperatures. This was not the case, and therefore it can be concluded that the majority of these compounds are formed from the tobacco decomposition. It is known they can be formed by pyrolytic decomposition of carbohydrates and tobacco structural polymers (e.g., cellulose, pectins and sugars) [28-31]. More recent studies have shown that pectin undergoes phase transformation between 150 and 180°C before an exothermic degradation [32,33]. The slow heating rate experienced by the tobacco inside the furnace may allow this type of thermal decomposition to occur to some extent, hence contributing to the measured levels.

For all six temperatures studied, three tobacco-specific nitrosamines or TSNAs (NNN, NNK and NAT) were quantifiable, but there was no consistent trend across the temperature range. NAB was found at 140°C and above. These nitrosamine compounds are known to be present in the tobacco leaf (Table 2) and can be directly transferred into cigarette smoke [1,34]. The fact that they were transferable at these low temperatures may be attributed to their structural similarities to nicotine and further similar thermochemical properties (e.g., the boiling point of NNN is 154°C at 0.2 Torr [35]). Pyrosynthesis of these nitrosamines have been reported in cigarette smoke [36] but the likelihood of this occurrence at the low temperatures in this work is low. As a possible explanation for the inconsistent yields, the possibility of analytical contamination was checked; during the LC-MS/MS analysis of the nitrosamines, blank solvent samples were run before and after as quality control samples; however, no contamination or carryover was found. For other compounds, such as acrylonitrile, HCN and phenols, the absence of detectable amount of these compounds in the emissions from the heated tobacco samples suggest that higher temperatures may be required for their formation.

**Table 2:** **Tobacco blend and rod physical parameters**

Tobacco rod length	mm	61.0
Total cigarette length	mm	83.0
Filter type*	Single section	Cellulose acetate
Tobacco weight	mg	598.0
Cigarette paper	Coresta Unit**	50
Unlit cigarette pressure drop	mm water gauge	54.0
Blend nicotine (dry weight basis)	%	3.25
Total sugar (dry weight basis)	%	14.5
*N*-nitrosonornicotine (NNN)	ng/gram of tobacco	97
4-(*N*-methylnitroso amino)-1-(3-pyridinyl)-1-butanone (NNK)	ng/gram of tobacco	43
*N*-nitrosoanabasine (NAB)	ng/gram of tobacco	12
*N*-nitrosoanatabine (NAT)	ng/gram of tobacco	151
Tobacco moisture	%	11.0
Cigarette ISO NFDPM***	mg	4.1

^*^The filter was removed from the tobacco rod for the heating experiments.

**Coresta unit is defined by the flow of air (cm^3^ min^−1^) passing through 1 cm^2^ surface of the paper at a measuring pressure of 1.00 kPa (cm^3^ min^−1^ cm^−2^ at 1kPa).

***NFDPM stands for “nicotine-free-dry-particulate-matter”; a convention used to compare yields made from machine-smoked cigarettes.

Heating tobacco from periphery at the temperatures used in this study significantly minimizes pyrolysis and prevents combustion reactions. Figure 2 also reveals a pronounced cooling effect caused by a puff, especially at the lower heating temperature (100°C). At the heater temperatures above 100°C, the thermal lag in the centre of the tobacco rod as demonstrated by the temperature plateau around 100°C was a visible feature (Figure 2) and linked to the energy required to release water. All of these are expected to influence the yields and relative proportionalities of the analytes measured in Table 1.

**Figure 3:**
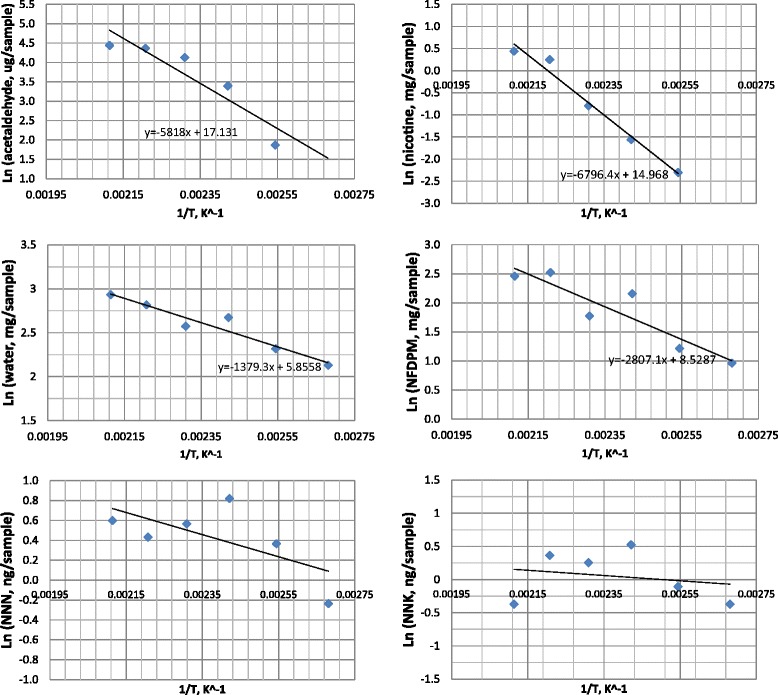
Arrhenius plots for the six analytes quantified in the heated tobacco aerosol.

In Figure 3, the yields of six analytes that were quantifiable under the full or majority of the temperatures were analysed using a pseudo-Arrhenius plot; the objective was to see whether their release could be empirically modelled to differentiate their release mechanisms. This is empirical because the tobacco rod could not be heated to the target temperature instantaneously when it was introduced into the furnace. The emission values plotted in Figure 3 were the results of accumulated release from a range of temperatures leading up to the set temperature. Hence this pseudo-Arrhenius approach cannot be used to calculate accurate kinetic parameters for the generation of these compounds, but comparison of relative trends between the compounds observed are valid. Reasonable linearity was seen in the pseudo-Arrhenius plots for acetaldehyde, nicotine, water and NFDPM values. As Table 1 shows, the measured TPM consisted of a large portion of water and a significant level of nicotine above 160°C. Thus, the calculated NFDPM captured the remaining condensed phase matter in the aerosol excluding these two compounds. A full analysis of the chemical composition of this condensed phase matter (NFDPM) is beyond the objective of this study.

Gradients obtained from linear regression in these plots gave the values for the pseudo-first order activation energy listed in Table 3. The value estimate for water, at 11.5 kJ mol^−1^ is about 3–4 times lower than the reference values for the enthalpy of vaporisation of water over the temperature range 100-200°C [37]. The enthalpy of vaporisation of pure nicotine of 53.3 (KJ mol^−1^) [38] is similar to the value estimated in this work, but previous thermal studies of nicotine evolution from the type of salts found in tobacco have identified effective activation energies for nicotine release of the order of 120 (KJ mol^−1^), consistent with bond breaking of the nicotine salt and evaporation of the unprotonated nicotine. This value is about 2.5 times higher than that estimated in the current work. Hence, the non-isothermal nature of the current experiment may be behind these underestimates. With this in mind, the effective activation energy for acetaldehyde release at 48.4 kJ mol^−1^, points to activation energies consistent with bond breaking rather than vapourisation, consistent with previous work showing it to be generated from thermal decomposition of sugars, pectin and cellulosic ingredients within tobacco [39]. The lower pseudo-activation energy for NFDPM of 23.3(KJ mol^−1^) measured in this work points to a mixture of evaporative and bond breaking routes. Poor linearity can be seen for NNN and NNK despite their structural similarities to nicotine. This might be attributed to their low levels in their emissions. In summary, the approaches described the potential to deduce useful information for different classes of compounds found in low-temperature heating of tobacco.

**Table 3:** **Pseudo-activation energies estimated from tobacco heating experiments and literature values**

**Compound**	**Pseudo-activation energy (KJ mol** ^**−1**^ **)**	**Enthalpy of vapourisation (KJ mol** ^**−1**^ **)**	**Literature value** [40] **(KJ mol** ^**−1**^ **)**
Water	11.5	36.3 ~ 40.7 between 100-200°C	-
Nicotine	56.5	53.3	-
Nicotine salts			115
Acetaldehyde	48.4	26.-27.6	-
NFDPM	23.3	-	-

From the emissions data obtained on the heated tobacco samples, estimates could be made of the degree to which the reservoir of the studied compounds within the tobacco rod were exhausted by the 7 puffs used in this study. This is shown in Table 4 for water, nicotine and three tobacco-specific nitrosamines. With the lowest boiling point among these compounds, the percentage of water released increased gradually with the increasing set heater temperature, to a final level of ca. 29% of the blend water content at 200°C. The percentage of nicotine released under the 7 puffs was only ca. 9% of the blend nicotine. The percentage released for the three TSNAs were even lower. Assuming first order kinetic processes, and using the integrated form of the rate equation, it was estimated that under the experimental conditions used the tobacco rod half life "times" would be of approximately 16 puffs for water, 50 puffs for nicotine and 2-300 puffs for TSNAs under 200°C heating conditions. Therefore tobacco heated to 200°C can generate emissions for a substantially longer period than a burning cigarette. This demonstrates the difficulty in setting a fixed puff number for the practical determination of its emissions from a low-temperature heated tobacco sample. Interactions between tobacco weight, tobacco composition, tobacco format, heating temperature, puffing parameters, etc., all contribute to the total emission levels.

**Table 4:** **Percentage of water, nicotine and three TSNAS released at the different temperatures against their levels in the tobacco sample**

	**Tobacco Heating Temperature (°C)**					
	**100**	**120**	**140**	**160**	**180**	**200**
Water released (%)	13	15	22	20	25	29
Nicotine released (%)	-	-	-	3	7	9
NNN released (%)	0.8	1.4	2.4	1.9	1.5	1.9
NNK released (%)	1.6	2.1	4.0	3.0	3.3	1.6
NAT released (%)	0.3	0.5	0.9	0.7	1.0	2.8

### Experimental

#### Tobacco heating device

**Figure 4:**
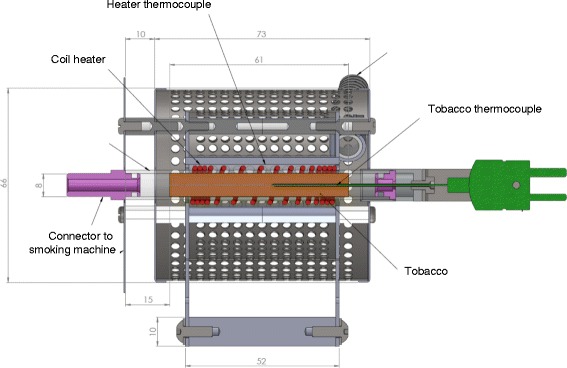
A schematic diagram of the tube furnace for low-temperature heating (the dimension shown are in mm).

Figure 4 is a schematic diagram of the tube furnace designed to accurately heat a quantity of cut tobacco (comparable to the amount used in a conventional cigarette) up to 400°C within ± 5°C. A separate AC-mains powered control unit was used to regulate the heat supplied to a wound wire heater. The furnace had a stainless steel casing with thermal shielding for safe handling. The outer dimensions were such that it was compatible with commercial linear smoke machines, allowing up to 10 devices to be puffed simultaneously to generate aerosol emissions. Tobacco samples in cigarette rod format were loaded from one end of the device (right, in Figure 4), hosted within the central segment of a 92 mm long quartz tube (10 mm outer diameter and 8 mm inner diameter). The tube had a stainless steel end-cap at one end (right, in Figure 4) and a connection tube on the other. The connection tube was approximately 30 mm long and of 8 mm outer diameter and 4 mm internal diameter. It fitted a Cambridge pad holder (a standard fixture used to trap particulate or aerosol). The stainless steel end-cap on the right had one centre hole (3 mm in diameter) that was designed to accommodate an end-piercing thermocouple to monitor the internal temperature of the tobacco being heated and also allowed airflow to be drawn through. Due to the fact that the furnace was mains-powered, the heating elements achieved a set temperature almost instantaneously; the actual heating rate for the tobacco sample was variable and determined by the heating time, given the identical quartz tubes and tobacco weights used.

#### Tobacco sample

The tobacco used in this study was already manufactured into a cigarette form (ca. 8 mm in diameter and 83 mm long, see Table 2), as this was thought to give a more consistent tobacco weight and density control along the length of the heating zone. Loose forms of tobacco or different materials may also be heated by this method. For each heating experiment, the filter of the cigarette was removed as its presence would have introduced the possible presence of filter components and thermolysis products into the aerosol. The remaining tobacco rod was inserted 15 mm from the connection end of the quartz tube. The furnace was then switched on and allowed to reach a set temperature before the loaded quartz tube was inserted into the heating zone. In this way, the heating profile of the tobacco sample was only determined by the sample. Once in position, the tobacco sample was heated for two minutes at the set temperature before puffing was initiated at the set temperature. A total of 7 puffs were taken for each sample – this was intended to mimic the duration of a cigarette being smoked (and was equivalent to the ISO smoking puff number of this cigarette; it was not intended to “exhaust the content” of the tobacco sample. The tobacco used in this study was a single grade Virginia type, designed for research purpose only. A number of physical and chemical parameters for the cigarettes were measured and provided in Table 2.

#### Aerosol chemical analyses

Chemical analyses of the aerosol emissions followed the procedures listed in Table 5. These are procedures developed for cigarette smoke analyses. At each temperature (100, 120, 140, 160, 180, 200°C), three replicates were taken for each heated tobacco sample using a single furnace with an unused tobacco rod. Each furnace was clamped into position and connected to the smoking port of a SM450 linear cigarette smoking machine (Cerulean, Milton Keynes, Bucks, MK14 6LY, UK). Puff parameters used were: 35 mL puff volume, 2 s puff duration, one puff every 60 s; this protocol was based on an ISO standard machine-smoking method [41].

**Table 5:** **Eight analytical methods used and their targeted analytes in aerosol**

**Analyte(s)**	**Aerosol collection**	**Analytical method**	**No of replicates**
Ammonia	Liquid impinger/0.05 M sulphuric acid	Ion chromatography (IC)	3
Carbon monoxide	Tedlar bag	Non dispersive infrared analyser	3
Nicotine, total particulate matter (TPM), nicotine-free-dry-particulate-matter (NFDPM), water	Cambridge filter pad	Gas chromatography (GC) with thermal conductivity for nicotine and GC-flame ionisation detection for water	3
Acetaldehyde, acetone, acrolein, butyraldehyde, crotonaldehyde, formaldehyde, methylethylketone (MEK), propionaldehyde	Liquid impinger/DPNH	Ultraviolet-high performance liquid chromatography (UV-HPLC)	3
Hydrogen cyanide	Liquid impinger/aqueous sodium hydroxide	Continuous flow analysis	3
Catechol, cresol (*o-*, *m- & p-*), hydroquinone, phenol, resourcinol	Cambridge filter pad	GC/MS	3
*N*-nitrosonornicotine (NNN), 4-(*N*-methylnitroso amino)-1-(3-pyridinyl)-1-butanone (NNK), *N*-nitrosoanabasine (NAB), *N*-nitrosoanatabine (NAT)	Cambridge filter pad	Liquid chromatography-Mass spectrometry/Mass spectrometry (LC-MS/MS)	3
Acrylonitrile, benzene 1,3-butadiene isoprene, toluene	Liquid impinger/chilled methanol, −77°C	Gas chromatography–mass spectrometry (GC-MS)	3

#### Thermogravimetric analyses (TGA)

TGA (PerkinElmer, STA 6000) were also performed on the tobacco to provide an overview of its thermal behaviour. For this experiment, between 13.4 to 13.8 mg of tobacco was loaded into an alumina crucible. For each TGA experiment, baseline temperature (30°C) was reached before the crucible with the tobacco sample was heated in air under 30, 40 and 50°C min^−1^ heating rates. These heating rates were broadly in line with that measured from the centre of the tobacco rod (see Figure 3); the heating rate experienced by the surface of the tobacco rod would be faster. Three replicates were run under each heating rate and averaged prior to interpretation. First derivative of the averaged weight loss (DTG) were also obtained. Prior to all the experiments, the tobacco samples were conditioned at 22°C and 60% relative humidity for at least 48 hr.

## Conclusions

In this study, we have developed an experimental method to generate emissions from tobacco samples that was heated between 100 and 200°C. This approach was applied to understand main thermophysical and thermochemical processes behind a number of compounds found in the aerosol generated by a smoking machine. For the majority of the compounds targeted, the temperatures were either too low for their presence in the aerosol or the amounts generated fell below the detection limits of the analytical methods used. For those compounds that were quantified

- Carbon monoxide, acetaldehyde, NNN and NNK were quantifiable at 140°C and 160°C, and crotonaldehyde and formaldehyde were quantifiable at 180°C and 200°C.
- Water, NFDPM, acetaldehyde and nicotine were found to obey pseudo-Arrhenius kinetics with increasing activation energy values. These appeared to agree with their known thermal release mechanisms, which ranging from evaporation, distillation to decomposition.

## Additional file

Additional file 1:
**Supplement Material - Toxicants in the test cigarette mainstream smoke.**
