# Supplementary material for: An experimental method to study emissions from heated tobacco between 100-200°C
Source: Chem Cent J. 2015 Apr 16;9:20. doi: 10.1186/s13065-015-0096-1 (PMC4418098; doi:10.1186/s13065-015-0096-1)
Supplement: Additional file 1: — Supplement Material - Toxicants in the test cigarette mainstream smoke. [file 13065_2015_96_MOESM1_ESM.docx]

### Supplement Material - Toxicants in the test cigarette mainstream smoke

####

The table below lists the mainstream smoke toxicant data obtained from the same cigarette with its filter attached, machine-smoked under the ISO standard smoking method [26]. They are provided for reference purpose only.

**Table - The level of toxicants (mean level ± SD, n=3) in mainstream cigarette smoke.**

| **Toxicant** | **Unit** | **Yield – Vents Open**  (mean±SD) |
| --- | --- | --- |
| Ammonia | µg/cig | 2.5 ± 0.4 |
| Carbon monoxide | mg/cig | 2.18 ± 0.13 |
| Nicotine | mg/cig | 0.62 ± 0.03 |
| TPM | mg/cig | 4.82 ± 0.19 |
| Water | mg/cig | 0.08 ± 0.07 |
| NFDPM | mg/cig | 4.12 ± 0.22 |
| Acetaldehyde | µg/cig | 119.8 ± 5.5 |
| Acrolein | µg/cig | 13.2 ± 0.8 |
| Crotonaldehyde | µg/cig | 3.7 ± 0.1 |
| Formaldehyde | µg/cig | 7.7 ± 0.7 |
| Hydrogen cyanide | µg/cig | 13.8 ± 1.2 |
| Catechol | µg/cig | 58.3 ± 4.4 |
| *m-*Cresol | µg/cig | 2.6 ± 0.2 |
| *o-*Cresol | µg/cig | 2.8 ± 0.2 |
| *p-*Cresol | µg/cig | 5.9 ± 0.5 |
| Hydroquinone | µg/cig | 50.6 ± 3.0 |
| Phenol | µg/cig | 15.0 ± 1.7 |
| NNN | ng/cig | 3.3 ± 0.4 |
| NNK | ng/cig | 4.5 ± 0.7 |
| NAB | ng/cig | 0.8 ± 0.1 |
| NAT | ng/cig | 9.8 ± 0.9 |
| Acrylonitrile | µg/cig | 2.2 ± 0.3 |
| Benzene | µg/cig | 15.2 ± 1.3 |
| 1,3-Butadiene | µg/cig | 14.4 ± 1.5 |
| Isoprene | µg/cig | 193.1 ± 21.2 |
| Toluene | µg/cig | 22.4 ± 2.9 |
| Acetone | µg/cig | 67.9 ± 2.2 |
| Butyraldehyde | µg/cig | 9.5 ± 0.5 |
| Methyl Ethyl Ketone | µg/cig | 15.6 ± 0.4 |
| Propionaldehyde | µg/cig | 11.9 ± 0.7 |
| Resorcinol | µg/cig | 1.3 ± 0.1 |
